# Social prescribing programs involving unpaid caregivers: A scoping review

**DOI:** 10.1371/journal.pone.0347299

**Published:** 2026-04-21

**Authors:** Zeest Kadri, Amal Khan, Maheen Raja, Jenna Smith-Turchyn, Jennifer Watt, Allison Williams

**Affiliations:** 1 Faculty of Health Sciences, McMaster University, Hamilton, Ontario, Canada; 2 School of Medicine, Toronto Metropolitan University, Toronto, Ontario, Canada; 3 School of Rehabilitation Science, McMaster University, Hamilton, Ontario, Canada; 4 Department of Medicine, University of Toronto, Toronto, Ontario, Canada; 5 School of Earth, Environment & Society, McMaster University, Hamilton, Ontario, Canada; Faculty of Health Sciences - Universidade da Beira Interior, PORTUGAL

## Abstract

Unpaid caregivers face significant health challenges due to the sustained demands of their roles. Social prescribing is a model of care that may help unpaid caregivers connect with non-medical community-based support systems to improve their health and well-being. Given the growing interest in social prescribing, there is a need to map out existing literature to understand its potential to support caregiver health. This scoping review describes the current landscape of social prescribing programs for unpaid caregivers. A protocol was previously published on Open Science Framework. We searched academic and grey literature from January 2000 to March 2025, following Arksey and O’Malley’s framework and JBI recommendations for scoping reviews. An inductive approach was used to analyse data in order to generate descriptive summaries and themes of program and outcome characteristics. We identified 17 studies for inclusion from 4376 titles and abstracts. Most programs were designed for both caregivers and care recipients to participate in together and involved a variety of self, provider and community-based referral pathways. Programs included nature, art, museum and physical activity-based experiences, and most were facilitated by a supervisor or coach. Program features varied, offering caregivers spaces to connect and socialize, strengthen their relationship with the care recipient, or express themselves creatively. Studies reported positive impacts on caregiver stress, the caregiver-care recipient relationship, and social connection. Logistical benefits such as low cost and accessibility, as well as challenges like transportation and scheduling were reported to influence program attendance and retention. While this review highlights the potential of social prescribing to support caregiver health, future research should explore opportunities to integrate programs within respite care, develop more structured referral pathways and evaluate the full social prescribing pathway in addition to virtual programming options to ensure effective implementation within broader health and social care systems.

## Introduction

Unpaid or informal caregivers play a crucial yet frequently overlooked role in healthcare systems worldwide, spending billions of hours providing essential care for individuals with chronic illnesses, mental health and behavioural conditions, disabilities and other age-related diseases requiring assistance with activities of daily living [1–4]. Health outcomes associated with unpaid caregiving are disproportionately negative, with many caregivers compromising their own financial, health, family and well-being needs to attend to their caregiving duties [5,6]. Evidence demonstrates that unpaid caregivers face an increased incidence of depression, burden, loneliness, as well as a decrease in quality-of-life [7,8,9]. Furthermore, the provision of unpaid care to individuals with varying conditions may give rise to distinct challenges such as stigma and the absence of condition-specific supports [10,11].

Shifting sociodemographic and cultural trends indicate that the proportion of unpaid caregivers around the world are expected to substantially increase over the next few decades, which is attributed to various factors such as the increase in aging populations, an increase in the rising prevalence of chronic or debilitating conditions such as dementia, as well as other work and healthcare system-related changes [12–15]. These trends and facts represent a significant global health challenge and necessitate that interventions, policies, programs and systems must work to evolve to address the complex needs of unpaid caregivers alongside care recipients. Beach and colleagues [16] argue that current policies and programs supporting unpaid caregivers around the world focus on relieving the burden associated with caring instead of considering the holistic needs of the caregiver that enable them to thrive in their caregiving roles. The demanding nature of informal caregiving presents unique challenges for the caregiver that must be addressed by helping them to find a balance between their own self-care while managing their caregiving responsibilities [17].

Social prescribing has emerged as an increasingly recognized model of care worldwide, linking individuals to non-medical community-based support systems aimed at improving health and well-being [18,19]. Social prescribing involves a navigational approach to care, and typically involves an element of referral by oneself, a healthcare provider or link worker, to a community or statutory service meant to attend to social well-being needs [20]. The first internationally recognized conceptual definition describes social prescribing as a ‘non-medical prescription to non-clinical supports and services within the community that is usually co-produced by an individual and a trusted individual in a community or clinical setting to subsequently improve health and well-being’ [21]. Today, social prescribing and navigational-based models of care are increasingly being implemented in different contexts across various parts of the world, both formally and informally [18]. In the United Kingdom where social prescribing has been formally integrated into the NHS for example, social prescribing can take the form of dance classes, hike excursions and more [22].

Allowing unpaid caregivers to participate in social prescribing programs may help to alleviate the disproportionately negative physical and mental health outcomes associated with their caregiving roles. This is especially important given that social prescribing models of care have the potential to address a wide spectrum of caregiving experiences and needs. While a growing body of literature highlights the promising impacts of social prescribing and explores caregiver perceptions regarding potential participation, there remains limited research on the availability, nature and effectiveness of social prescribing programs for this population [23–26]. Furthermore, many existing caregiving interventions or programs focus on improving caregiving competencies rather than providing an avenue to facilitate the overall well-being for this population. This gap in the literature warrants a preliminary investigation of the current landscape regarding social prescribing among this population, especially as social prescribing and related navigational models of care continue to be evaluated and piloted in the literature [18].

We aim to collect and report on the growing body of literature related to answer the following research question:

What are the current practices, outcomes, benefits, and challenges of social prescribing programs involving unpaid caregivers?

The results of our scoping review will help to map out the existing evidence associated with social prescribing programs for unpaid caregivers and allow for the identification of evidence gaps where further research is needed.

## Methods

A protocol was created and published on Open Science Framework in 2024 [27].

Given that social prescribing is an emerging model of care that is currently being adopted in different capacities and contexts around the world, a scoping review was determined to be the most appropriate methodology to broadly address relevant aspects of the research question. This proposed scoping review followed framework and reporting guidelines as established by Arksey and O’Malley [28] in addition to the updated JBI methodology for scoping reviews, respectively [29,30]. To ensure transparent and complete reporting, the Preferred Reporting Items for Systematic Reviews and Meta-Analyses Extension for Scoping Reviews (PRISMA-ScR) checklist [31] was also used, which may be accessed in S1 Checklist.

### Eligibility criteria

In accordance with our protocol, this scoping review aimed to identify and present the available English-language academic and grey literature published between January 1, 2000 till June 26, 2024. This limit was selected to ensure relevance to the subject topic, as social prescribing is a newer and developing concept which gained popularity after the year 2006 [32,33]. The search was limited to English-language publications due to various resource constraints related to availability of translation services, and the study team acknowledged that this may have potentially resulted in the exclusion of literature that would impact the generalizability of findings. An updated search was carried out on March 30, 2025.

Articles were included if they involved an unpaid caregiver participating in a specific social prescribing program or service. For this scoping review, we used the inclusive operationalized definition of social prescribing described by Muhl and colleagues [21]. This allowed for the creation of inclusion criteria that was informed by the Population, Concept and Context framework [29], as seen in Table 1.

**Table 1:** Evidence Selection using the Population, Concept, Context Framework.

**Population** • Caregiver (e.g., family, friend, neighbour, colleague, community member) who provides unpaid care for individuals within a community setting **Concept** • Social prescribing program involving an element of recruitment or referral to non-clinical supports within a community-setting that aim to improve an individual’s health and well-being **Context** • Study conducted and published between January 1, 2000 and March 30, 2025 • Publication in the English language • Any relevant primary publication from around the world

Studies that did not actively involve caregiver participation, did not discuss caregiver outcomes, or evaluated caregiver needs without actual implementation of a social prescribing program were excluded. Conference papers and related abstracts were excluded, as well as any non-English literature and reviews. Backward reference searches of included studies were also completed to identify any other full-text studies for inclusion.

### Information sources

An initial search was conducted on Ovid MEDLINE Ahead of Print to identify relevant keywords and index terms on the proposed topic. Furthermore, other terminology associated with social prescribing was identified using Google. This allowed for the creation of a larger search strategy, which was informed by an experienced librarian at McMaster University in Hamilton, Canada. The search strategy with the identified keywords and index terms were appropriately adjusted for each database or information source utilized. A sample of a full search may be accessed using S2 File.

Databases used to identify published studies included Social Science Abstracts, Social Work Abstracts, AgeLine, CINAHL, Emcare, Global Health, OVID Medline Ahead of Print, APA PsychInfo and Web of Science. Furthermore, Google Scholar was utilized to identify other relevant studies. Google Advanced Search was used to identify unpublished or grey literature, including dissertations and reports in countries where social prescribing has been most prominently implemented and/or studied according to the literature (e.g., United Kingdom, Australia, Canada, United States) [18]. Combinations of keywords used for the grey-literature search included ((social* prescri*) AND (caregiv* OR carer*)). Requests for other relevant keywords or grey-literature sources were also discussed with a senior research project manager at the Canadian Institute for Social Prescribing (CISP) to ensure a comprehensive overall search.

### Selection of full-text articles

All identified citations and their associated full-text PDFs were uploaded into Covidence [34]. None of the automated features on Covidence were used. After removal of duplicate studies, pairs of independent reviewers (ZK, AK, MR) identified relevant studies for full-text inclusion by utilizing title and abstract data. For available grey-literature, associated PDFs were also uploaded manually into Covidence and screened.

Upon preliminary evaluation of 161 studies that made it to full-text review, it was noticed that some social prescribing pathways involved referrals to multiple unspecified community programs. Such studies were excluded because program characteristics could not be adequately explored. The research team also agreed to exclude any knowledge-based, behavioural therapy-based or virtual programs in order to maintain focus on socially prescribed models of care within community settings, which was seen to be emphasized in the literature [21]. Additionally, studies with no clear element of recruitment or referral into a specific program/service were excluded. These types of programs were excluded because they were deemed to be distinct from the overall aims of social prescribing according to the initial operationalized definition that we used to inform our inclusion criteria. These restrictions were applied to ensure clarity and rigor in identifying full-text studies for selection.

Any potential disagreements in full-text screening were resolved through detailed discussion or alongside an additional reviewer on the research team (AW). No critical appraisal of evidence was undertaken.

### Data extraction and data synthesis

Two reviewers (ZK, MR, AK) independently screened important details on study characteristics and outcomes in duplicate using Microsoft Excel. Important and relevant information related to the sociodemographic characteristics of the unpaid caregiver as well as elements of the social prescribing program were extracted for all studies [30]. To align our data extraction with our research question, we utilized thematic analysis to report on outcomes, logistical benefits, and logistical challenges of the program [35]. An inductive approach was used to analyze extracted data from included studies, allowing for the creation of appropriate subthemes that would be presented using descriptive summaries. Data analysis involved multiple meetings between two or more members of the review team to finalize study themes and subthemes (ZK, AW, AK, MR).

## Results

**Fig 1:**
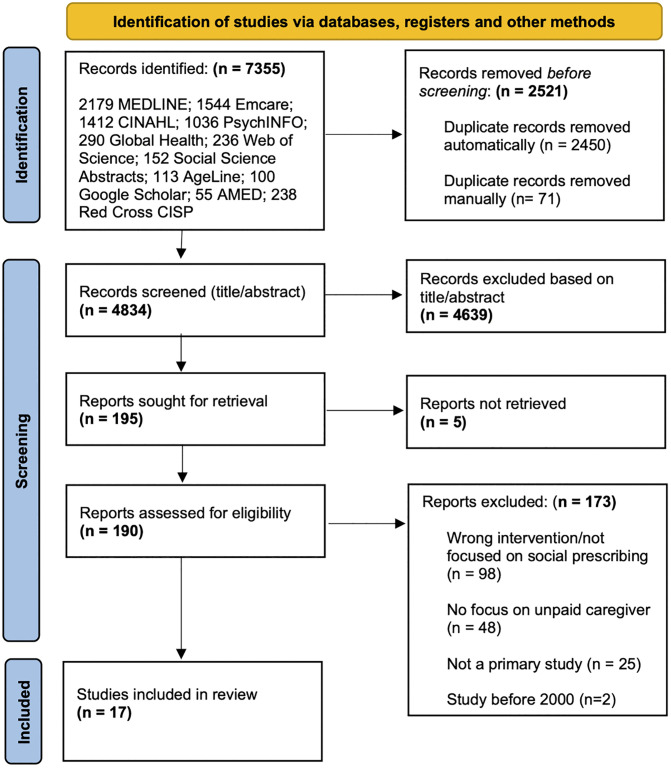
PRISMA 2020 Flow Diagram.

Following title and abstract screening of 4376 records, a total of 17 studies were selected for inclusion (Fig 1).

### Characteristics of included studies

Table 2 contains a summary of relevant study and demographic characteristics.

**Table 2:** Characteristics of Included Studies.

Study Author, Year	Country of Study	Methods	# of CGs	Age of CGs* (years)	% Female CGs	CG Race/Ethnicity	Relationship of CG to CR	CR Health Status/Condition
Baik et al. 2024	Republic of Korea	QN: Randomized control trial	N = 60 (30 exp, 30 ctrl)	42.66 (SD 4.25)	NR	NR	100% parental figures to CR	Atopic Dermatitis
Baker et al. 2018	Australia	MM: Quasi-experimental and interviews	N = 14 (8 in exp, 6 ctrl)	72.29 (6.72)	64.2%	NR	78.6% spouses, 14.3% adult children, 7.1% sibling to CR	Dementia
Burnside et al. 2017	United States	QL: Semi-structured interviews	N = 21	NR	NR	5% African American, 5% Asian, 90% White	52% spouses, 48% children to CR	Dementia
Donnelly et al. 2021	Canada, United States	MM: Pre-post with open-ended data	N = 152	49 (SD 13.1)	84.90%	NR	NR	Traumatic Brain Injury
Dorard et al. 2022	France	QN: Pre-post	N = 117	Overall: 12 (range 7–17) Children (9.8, range 7–12) Adolescents (mean 13.9, range 13–17)	71.80%	NR	100% youth and adolescent Children to CRs	45.3% of CR had somatic disease, 18.8% disability, 5.1% mental disorder, 34.2% of CGs did not know name of CR’s condition
Fancourt et al. 2019	United Kingdom	QN: Quasi-experimental	N = 62 (33 exp, 29 ctrl)	Ctrl Group: 51 (SD 15) Exp Group: 58 (SD 14)	77.4%	NR	Spouse, relative, close friend	Cancer
Flatt et al. 2015	United States	QL: focus-group interviews	N = 10	6 CGs 60 or older, (NI for remaining 4)	60%	NR	Spouse, child	Early-stage Alzheimer’s (n = 8) or related cognitive disorders (n = 2)
Giebel et al. 2021	United Kingdom	QN: pre-post	N = 11	74 (range 57–90)	63.60%	NR	90.9% spouse 9.1%, children	Dementia
Girdler et al. 2024	Canada	QL: semi-structured interviews	N = 6	Range: 60–79	NR	NR	Spouses	Dementia
Lee et al. 2022	Ireland	QL: semi-structured interviews	N = 4	Range 30–79	75%	NR	NR	Dementia
McGuigan et al. 2015	New Zealand	QL: focus-group interviews	N = 7	Range 35–55 + years	84%	NR	71.4% spouses, 28.6% children	Dementia
McManus et al. 2022	United States	MM: pre-post and semi-structured interviews	N = 32	6.3% aged 41–50, 18.8% aged 51–60, 28.3% aged 61–70, 28% aged 71–80, 18.6% aged >80	84.4%	Race: 84.4% White, 15.6% other Ethnicity: 6.3% Hispanic, 90.3% Non-Hispanic	78.1% spouses, 18.8% children (daughters), 3.1% sibling (sister)	Dementia
Mittelman and Papayanno-poulou, 2018	United States	MM: pre-post and focus groups	N = 11	71.7 (SD 8.3)	54.54%	100% Caucasian	81.8% spouse/partners, 9.1% adult child, 9.1% close friends	Dementia
Pienaar and Reynolds, 2015	United Kingdom	QL: interviews	N = 8, only 4 interviewed	Range 50–79	75%	100% Caucasian	75% spouses, 25% adult children	Dementia
Razani et al. 2018	United States	QN: Randomized-control trial	N = 78 (n = 28 independent prescription, 50 in supported prescription)	37% aged 18–34, 36% aged 35–44, 27% aged 45 and older	58.30%	67% African American, 5% Non-Latino White, 15% Latino, 13% Other	100% parents	Children with: ADHD, depression, anxiety, autism spectrum disorder, developmental delay, asthma, obesity, other
Sun et al. 2022	Canada	QL: semi-structured interviews	N = 13	74.35 (range 63–83)	58.3%	NR	NR	Dementia
Vaajoki et al. 2023	Finland	QL: thematic interviews	N = 12	Over 65	NR	NR	100% spouses	Stroke, memory disorder or neurodegenerative diseases

*Age of unpaid caregivers all reported as mean unless otherwise stated.

CG: caregiver; CR: care recipient.

exp: experimental; ctrl: control.

QN: quantitative; MM: mixed-methods; QL: qualitative; NR: not reported.

ADHD: attention-deficit/hyperactivity disorder.

Of the 17 studies selected for inclusion, eight (47.06%; 8/17) were published between 2014–2019 [36–43] The remaining nine studies (52.94%; 9/17) were published between 2021–2024 [44–52].

With regards to study methodology, most studies were qualitative, utilizing interviews and focus groups to better understand an unpaid caregiver’s participation in a social prescribing program (47.1%; 8/17) [37–40,42,48,49,51,52]. Five studies were quantitative (29.4%; 5/17) [38,43,44,46,47], and four studies involved mixed methods (23.5%; 4/17) [36,41,45, 50].

Five studies were published and conducted within the United States (29.4%; 5/17) [37,39,41,43,50], with three done in the United Kingdom (17.6%; 3/17) [38,42,47], two in Canada (11.8%; 2/17) [48,51], one in Australia (5.9%; 1/17) [36], one in Finland (5.9%; 1/17) [52], one in France (5.9%; 1/17) [46], one in Ireland (5.9%; 1/17) [49], one in New Zealand (5.9%; 1/17) [40], and one in the Republic of Korea (5.9%; 1/17) [44]. Furthermore, one study was conducted in a multiple-country setting, involving both Canada and the United States (5.9%; 1/17) [45].

### Demographic characteristics of unpaid caregivers involved in social prescribing programs

The age of the caregivers varied across studies; given this variation, a mean age was not deemed appropriate to tally. Though all studies included adult caregivers, only one study involved youth caregivers under the age of eighteen [46]. In all studies that reported the sex of the unpaid caregiver (76.5%; 13/17) the majority (more than 50%) were female [36,38–43,45–47,49–51].

The relationship between the unpaid caregiver to the care recipient also varied across the studies. Two studies (11.8%; 2/17) involved parental caregivers of children with varying conditions [43,44]. Most included studies included caregivers of people with dementia or related cognitive disorders (64.7%; 11/17) [36,37,39–42,48–51].

### Current practices of social prescribing programs involving unpaid caregivers

As indicated in Table 3, a variety of social prescribing programs were offered for unpaid caregivers to participate in. Two studies (11.8%; 2/17) involved nature-based prescriptions, and both these programs were designed for parental caregivers and their children to participate in together [43,44]. Four studies (23.5%; 4/17) involved recreational physical activity, including yoga or other movement-based programming for the unpaid caregiver and care recipient to participate in together [45,47,48,51]. A variety of art-based programs were also reported; four studies (23.5%; 4/17) involved musical programs encompassing choir singing or songwriting [36,38,41,49]; three (17.6%; 3/17) involved museum/gallery-based programs [39,40,42]; and four (23.5%; 4/17) involved other arts based programming [37,46,50,52]. Program duration time varied considerably across included studies, ranging from single-session activities to multi-session programmes delivered over a period of multiple weeks. The majority of included studies were reported to deliver weekly sessions, and one study only featured a one-time activity facilitated over three hours [39].

**Table 3:** Components of Social Prescribing Programs Involving Unpaid Caregivers.

Name of Program	Program Description	Mode of Referral/Involvement of CGs	Duration	Facilitator	Dyadic Participation? (Y/N)
Therapeutic Gardening Program	Horticultural and gardening activities with physical activity in a public community garden; designed to foster active interaction and communication between children and caregivers, reduce stress and increase parenting efficacy and work to improve caregiver’s quality-of-life	**Self-referral**: posters or online bulletin boards in hospital/health centres	15 sessions over approx. 4 months	NR	Y
Songwriting Program	Group-based program to allow CGs to write, rehearse and perform their own songs to share and express their caregiving experiences	**Community-referral:** day-care centers	6 classes, 1 hour/week	Trained music therapist	N
*here:now* Program	Group-based program involving a monthly discussion-based gallery tour as well as an art-making experience to optimize care, support mood and social engagement	**Primary-care and community referral:** physicians, Alzheimer’s Association, and other community sources.	Ongoing program that includes one-time tours and six-week classes.	Trained museum educator, teaching artist	Y
*LoveYourBrain Yoga* Program	A 6-week, community-based yoga with psychoeducation program to improve quality of life, promote community reintegration and build skills in resilience	**Primary-care, community and self-referral:** family, friends, healthcare professionals, social media, support group, yoga studio	6 weeks	Experienced yoga teachers	Y
Cinema Program	Cinema-related artistic creation, games, outings, leisure activities and discussions to express and share caregiving experiences	**Primary-care, community and self-referral:** various partners from health, social, and school sectors, or by families	2 weeks	Cinema and drama professionals	N
Choir Singing Intervention	Choir sessions led by professional choir leader to sing and learn choir music, with time allocated for socialization to foster positive emotion, social support and reduce fatigue and stress	**Primary-care, community and self-referral:** National Health Service (NHS) hospital trusts in Greater London, carer support groups, community and charity events, social media	12 weeks	Professional choir leader	Y
Art Museum Engagement Activity	Museum engagement activity with a guided tour followed by an art making activity	**Community-referral:** Alzheimer’s Disease Research Center (Pittsburgh)	3-hour activity, only took place once	Trained facilitator	Y
*Happy and Healthy* classes	Physical and mental well-being exercises that include low-impact physical activity, local walks, Tai Chi, relaxation techniques, mindfulness, and games to impact overall well-being	**Primary-care or self-referral:** psychiatrists at memory clinics, general practitioners, dementia care navigators or self-referred via word of mouth and social media.	1-hour weekly classes, (ongoing)	Community center advisor	Y
*Minds in Motion® (MiM)* Program	Program that involves age-appropriate physical activities and mentally stimulating activities that foster social connection and mentally stimulating activity	**Community-referral:** local Alzheimer’s Society	2-hour weekly sessions	Program coordinator	Y
Community-based Group Singing Program	Flexible music-based singing program where participants played an active role in suggesting songs and music genres to learn and sing to promote social connection, expression and creativity in the moment	**Primary-care referral**: University teaching hospital memory service	6 weeks	Music therapist	Y
Museum Visit Program	Community-based Museum activities, discussions and tours with opportunities to handle objects, view images and visit galleries to facilitate socialization, provide a positive and shared experience	**Community referral:** Alzheimer’s Aukland	6 sessions over 6 weeks	Museum volunteer guide	Y
Multi-modal Performing Arts Program	Weekly performance-based activities, with a combination of theatre games, movement exercises, improvisation, and/or scene work.	**Primary-care and self-referral:** physician, or through community events/websites	8 weekly classes	Trained facilitators	Y
*The Unforgettables* Community Chorus Group	Vocal and performance techniques taught by conductors during rehearsal sessions to improve quality of life and social connectedness via shared participation in an enjoyable social activity	**Community-referral:** existing support group/ support group representatives, existing community program collaborators	13 weeks	Music therapist and conductor	Y
Creative Arts Leisure Program	Artmaking and a visit to local art in one gallery session, with flexibility allowing for participants to choose what arts and crafts they would like to work on to express their experiences in words or through art.	**Community-based and self-referral**: existing social café	5 weeks, 1.5 hours/week	Arts tutor	N
*Stay Healthy In Nature Everyday: SHINE*	Park prescription and organized group family outings to improve stress and other behavioural and health outcomes	**Primary-care referrals:** from healthcare providers	Weekly visits, with monthly family outings	NR	Y
*Living Well With Dementia:* Recreational Programs	Multiple social recreational programs involving physical activity and socialization (1) Minds in Motion; 2) Brain Wave Café; and 3) Caregiver Support Groups.) that are facilitated by community volunteers to promote sense of social connectedness	**Community and self-referral:** existing community programs that had a primary referral involvement (e.g., Minds in Motion; Brain Wave Café and Caregiver Support Group)	NR	volunteer	Y
*Joy of Arts*	Various music, creative dance and visual art sessions to enhance well-being	**Community-referral:** Local association	12 weeks, 45 minute sessions	Music, dance and art pedagogues	Y

CG = caregiver.

CR = care recipient.

Most social prescribing programs (76.5%; 13/17) were designed for both unpaid caregivers and care recipients to participate in together. Unpaid caregivers in three studies participated in a program without the care-recipient, and this was typically done by offering the unpaid caregivers some type of respite service while they participated in the programming [36,42,46]. One study involving young children caregivers offered the program during their holidays [46].

With regards to the referral pathway involved, most studies involved a combination of self-referral, community and/or primary-care referral pathway to facilitate participation in the social prescribing programs. Community referrals were seen mostly through the use of day-care centers or local associations, and primary-care pathways usually involved a healthcare provider such as a physician. Primary care referrals as a method of referral by itself was described in only two studies [43,49].

### Program features

Though all programs that were offered to caregivers met the formal definition of social prescribing according to our inclusion criteria, there were some program features that were commonly observed among studies. Firstly, all programs in the included studies were designed to foster well-being; none were designed to promote skill-development or improve caregiving knowledge/caregiving competencies.

Programs that involved an art-based component (64.7%; 11/17) were observed to prioritize a space for the caregiver to express themselves or their caregiving experiences in a supportive environment with their care recipients and other caregivers [36–42,46,49,50,52]. Programs that involved a physical activity component (23.5%; 4/17) were observed to mostly be drop-in programs where the caregiver and care recipient were able to arrive at their convenience; these programs did not have any end goal in mind and were facilitated to be a space for the caregiver and care recipient to engage in activities together [45,47,48,51]. Furthermore, programs that involved an outdoor space for caregivers and care recipients (11.8%; 2/17) were designed to promote active interaction and communication and well-being [43,44]. Most programs, regardless of type, were facilitated by trained facilitators or coaches. Only two programs (11.8%; 2/17) involving outdoor, hands-on activities such as a park prescription program and an outdoor therapeutic garden, were not reported to involve formally trained individuals supervising the social prescribing program [43,44]. Among studies that prioritized caregiver participation without the dyad and were conducted over a fixed-time period, the caregivers were often working towards some end-goal such as creating a film or creating and performing a song [36,46]. These additional program features are reflected in Table 3.

### Outcomes

Seven studies (41.2%; 7/17) utilized quantitative outcome measurements relating to the unpaid caregiver’s physical and mental health after participation in a social prescribing program [36,41,43,44,46,47,50]. Among these studies, commonly reported mental health outcomes included depression (42.8%; 3/7), quality of life (28.6%; 2/7), burden (28.6%; 2/7) and stress (28.6%; 2/7) using a variety of scales [36,41,44]. All quantitative studies reported improved caregiver mental and physical health related outcomes, both significant and non-significant.

In accordance with our research question, we further organized other reported qualitative outcomes for unpaid caregivers. These reported outcomes were related to caregiver stress, the relationship between the caregiver and care-recipient, as well as experiences of social connection with other caregivers in similar situations after participation in a social prescribing program.

### Caregiver stress

A common theme observed among seven studies (41.2%; 7/17) was the ability of the socially prescribed program to positively impact the unpaid caregiver’s stress levels associated with caregiving [37,38,42,45,46,51,52]. For example, unpaid caregivers involved in a choir singing program recalled that they were able to temporarily escape from everyday stressors and challenges associated with caring for a loved one through participation [38].

Despite the ability of the program to mitigate stress, a few studies reported stressors associated with other aspects of programming. For example, it was noticed that in two studies where only the caregiver was involved in the social prescribing program without the presence of their care recipient, there was some element of worry or concern from the caregiver’s end as they were not physically there for the care recipient [42,46]. In one study, there was some stress reported by young caregivers related to participation in a Cinema Arts program because they needed to complete their creative projects within a certain time frame [46].

### Relationship between caregiver and care recipient

A total of eight studies (47.1%; 8/17) reported on how the social prescribing program impacted the relationship between the caregiver and care recipient [37,43–45,48–50,52]. Programs where caregivers and care recipients participated together allowed relief from caregiving duties while affirming the relationship between the caregiver and care recipient at the same time [37]. In a quantitative study, the relationship between the caregiver and care recipient was observed through a self-reported increase in park visits [43].

More specifically, two studies addressed how participating in a program jointly allowed for the unpaid caregiver to better understand the challenges faced by the care recipient. One caregiver described how the program allowed for the caregiver and care recipient to interact outside of their everyday roles, allowing them to see beyond their dementia diagnosis [50]. In another study, unpaid caregivers participating in a yoga program reported increased compassion for their care recipients as they understood the challenges faced by them [45].

### Social connection with other caregivers

We identified a total of 11 studies (64.7%; 11/17) that addressed opportunities for social connection with other caregivers [36,39–42,46,48–52]. In particular, these studies reported on how caregivers were able to interact with other caregivers in similar situations like themselves and share experiences with each other. For example, caregivers involved in a songwriting program discussed how they were able to establish a sense of group belonging with other caregivers due to the fact that the program allowed them to creatively express their caregiving experiences that other programs were not able to fill [36].

### Benefits and challenges of social prescribing programs

Eleven included studies (64.7%; 11/17) reported certain benefits and challenges related to the logistics and implementation of social prescribing programs in the community for the unpaid caregiver (37, 39, 42, 43, 44–47, 49–51). It was identified that many programs provided appropriate accommodations for the caregiver through accessible transport options, small group settings and waived or discounted fees which facilitated their participation. One study also identified that the small group setting, as well as the involved museum staff, was a benefit to their participation in a museum-based program [39].

Common challenges reported in three included studies included transportation [45,47,51], as well as scheduling conflicts that prevented consistent attendance to the program [39,50,51]. Additionally, one study identified that because of the care recipient’s condition, some caregivers did not attend because they did not think the program was a good fit for them [50].

## Discussion

This scoping review synthesizes a growing body of knowledge by describing current reported practices, outcomes, benefits and challenges of community-based social prescribing programs involving unpaid caregivers. Our review indicates that this is a rapidly evolving field of knowledge, especially since a large proportion of included studies have been published within the last five years in different countries around the world. According to our results, a variety of arts-based, physical activity and nature-based programs are currently and commonly provided to unpaid caregivers, with most programs designed and offered for both caregivers and care recipients to participate in together. In contrast, very few programs were offered to the unpaid caregiver as respite; this is an avenue worth exploring for future research to integrate social prescribing programs for unpaid caregivers, especially since many respite programs take place in community-based settings such as day-care centers. This may help to move respite care from simply a break from caregiving, whereby more structured opportunities for the caregiver are provided to form social connections and engage in activities that promote well-being, as tailored towards the caregiver’s needs.

Our findings also indicate that methods of referral or entry into social prescribing programs for this population varied considerably across studies. We observed that a combination of self-referrals, community-based referrals and primary-care referrals were commonly used. This variability in referral strategy suggests that although access to social prescribing is broad and wide-ranging, it may also be fragmented, informally adopted and difficult to navigate, especially for those who are older or unfamiliar with available services. These observations are reflected in the literature, especially as unpaid caregivers and their care recipients consistently demonstrate a need for embedded community-based resources that are flexible and accessible and led by trained staff, such as specially trained care-coordinators [53,54]. Furthermore, many formalized social prescription programs around the world utilize specialized link-workers, family navigators or liaisons, and this may help unpaid caregivers to access resources they need in community-settings more efficiently. The use of these roles or individuals in future programs may provide more structured pathways for unpaid caregivers and their care recipients to access community-based resources more efficiently and may also help to mitigate negative health outcomes [53].

With regards to the outcomes observed among unpaid caregivers, our review found that social prescribing programs in the community described positive outcomes on the physical and mental well-being of unpaid caregivers as well as on their social well-being. These findings are consistent with existing literature, as social prescribing and other psychosocial programs have positive outcomes on the well-being of individuals and older adults [55–57]. We also observed that common challenges experienced by unpaid caregivers were reported to be related to transportation and scheduling conflicts. This highlights the importance of designing social prescribing programs that are adaptable to the needs of caregivers, potentially by use of participatory and collaborative research and flexible program design. For example, addressing issues such as scheduling conflicts through drop-in or hybrid formats may overcome both transport and scheduling issues.

Our observations throughout the research process offer other important insights in this emerging area of study. Throughout the screening process, it was found that a large proportion of records that made it into full text review were ultimately excluded because the unpaid caregiver did not actively participate in the social prescribing program, as the intervention primarily target care recipients. In these studies, unpaid caregivers were only mentioned in terms of their role in facilitating the care recipient’s participation, such as transporting or assisting them to access socially prescribed programs. Even in some studies that involved active participation of unpaid caregivers in social prescribing programs, there appeared to be a lack of analysis or results that specifically pertained to the unpaid caregiver’s own health and well-being. This may indicate that unpaid caregivers are often sidelined and positioned as secondary participants in research primarily designed for care recipients, and that programs may work to offer them respite to ease their duties rather than focus on the overall well-being of the caregivers themselves. Even though dyadic programs benefit both parties, we also advocate for the design of caregiver-centred models of care. These findings underscore a significant gap in ongoing research and practical initiatives, pointing to a better need for future studies to address the health outcomes of this population and recognize their roles as caregivers rather than only extensions of the care recipient.

Another observation worth consideration throughout the research process was the fact that all studies marked for inclusion were not necessarily described as conventional “social prescribing” programs or used that specific term. Social prescribing is a relatively new and emerging concept and term in modern research that is still being implemented in informal and formal contexts worldwide [18]. Due to this reason, the terminology used to describe these programs may vary significantly across different studies and settings. Throughout the development of the protocol, efforts were made to try to use a broad search strategy. This was done through consultations with librarians, project managers with the Canadian Institute of Social Prescribing, as well as existing literature. During the screening process, efforts were made to map and identify an element of recruitment or referral into community-based programs that involved unpaid caregivers, even if the term “social prescribing” was not explicitly mentioned. As such, if the referral or recruitment pathway of the unpaid caregiver was not clear in the study manuscript, then a potentially eligible study would be excluded. This ensured that there was methodological rigor throughout the screening process to capture a diverse range of community-based programs and initiatives that aligned with the core principles of social prescribing for unpaid caregivers, even when different terminology outside of the specific term “social prescribing” was used. We also excluded virtual programs to focus on in-person, community-embedded supports involving social interaction. However, we acknowledge that this may limit relevance given the growing role of virtual social prescribing initiatives.

Furthermore, as this scoping review involved caregivers across various ages and backgrounds, future studies could also work to better understand the intersectional needs of specific caregiver populations. We found that our evidence base mostly included informal caregivers of people living with dementia, which is also dominant in caregiving research. Being able to expand to more diverse caregiving contexts could help to design tailored programs for caregiving for individuals with different conditions. As such, we suggest expanding programs to diverse caregiving contexts in order to clarify whether program design is better if it is condition-specific or more broadly applicable.

Additionally, research clearly demonstrates that outcomes related to unpaid caregiving are influenced by various intersecting factors such as race, ethnicity and gender [58–60]. Future studies should not only stratify and analyze results according to different health equity factors, but also consider how various structural inequities influence access to and benefit from socially prescribed programs. For example, since most participants in reported studies were women, it is unclear if the programs were gender responsive. These considerations are crucial given the gendered distribution of unpaid caregiving and as such, must be supported in future program design.

Our review has limitations. First, due to the nature of scoping reviews, the studies included in this review were of varying methodologies, with many being qualitative studies that involved focus groups or interviews. This makes it difficult to quantify or see definite and conclusive trends associated with the benefits or challenges of social prescribing programs for this population. It also elucidates the need for more quantitative studies with control groups as well as systematic reviews to measure quantifiable changes and trends in unpaid caregivers’ health and well-being moving forward while assessing the risk of bias and quality of the evidence included. We also push for more quantitative research to better understand mechanisms of effectiveness on health and well-being. Social prescribing may also be conceptualized as a complex intervention and future research may benefit from appropriate frameworks to systematically evaluate the effectiveness of such programs [61,62].

Additionally, the scope of this review only represents one component of the larger social prescribing pathway and does not capture the entirety of the whole social prescribing process such as link referrals, assessment of needs, and evaluation of program participation among caregivers. Future studies could work to assess the entirety of this pathway and fill in existing gaps in knowledge to provide a more holistic understanding on how social prescribing operates in practice, which will also help bridge gaps or inefficiencies that may exist within the system. In our review, we also did not include knowledge-based, home-based or virtual programs, and this may have excluded a range of relevant programs that could be considered as socially prescribed programs. However, the importance of virtual programs such as virtual support groups for example, may be relevant and promising and may overcome barriers to accessibility, especially in a post-pandemic era. Future research efforts should explore how different models of social prescribing can be leveraged using digital platforms or methods of telehealth to improve accessibility for unpaid caregivers and their care recipients.

## Conclusion

Overall, this scoping review offers valuable insights into the current landscape of social prescribing for unpaid caregivers, emphasizing their potential benefits for health and well-being while also describing certain challenges that they face in access and participation. Our findings may help to inform future research and guide the development of inclusive and successful programs to better support a population that is often overlooked in health services planning. Unpaid caregivers require meaningful support and resources to address their own health needs, and social prescribing holds the potential to be a transformative rather than supportive process that reimagines how communities and health systems recognize, value and respond to the needs of this population.

## Supporting information

S1 Checklist PRISMA Extension for Scoping Reviews Checklist. (DOCX)

S2 File Sample Search Strategy. (DOCX)

## References

[1] Phillips R, Durkin M, Engward H, Cable G, Iancu M. The impact of caring for family members with mental illnesses on the caregiver: a scoping review. Health Promot Int. 2023;38(3):daac049. doi: 10.1093/heapro/daac049 35472137 PMC10269136

[2] Roth DL, Fredman L, Haley WE. Informal caregiving and its impact on health: a reappraisal from population-based studies. Gerontologist. 2015;55(2):309–19. doi: 10.1093/geront/gnu177 26035608 PMC6584119

[3] Schulz R, Eden J, National Academies of Sciences, Engineering, and Medicine. Family caregiving roles and impacts. Families caring for an aging America. National Academies Press (US); 2016. 27905704

[4] Smith M, Kuretich C. Informal caregiving: Measuring the cost and reducing the burden. SOA Research Institute. 2023 Apr. Available from: https://www.soa.org/resources/research-reports/2023/informal-caregiving-reducing-burden/

[5] Campbell C, Walker J. Informal caregiving and the risk of material hardship in the United States. Health Soc Care Community. 2022;30(5):e1701–10. doi: 10.1111/hsc.13597 34617649

[6] Greenwood N, Mezey G, Smith R. Social exclusion in adult informal carers: A systematic narrative review of the experiences of informal carers of people with dementia and mental illness. Maturitas. 2018;112:39–45. doi: 10.1016/j.maturitas.2018.03.011 29704916

[7] Boyle D. Caregiving within the context of elder care. Informal caregivers: from hidden heroes to integral part of care. Springer International Publishing. 2023. p. 33–67.

[8] Grycuk E, Chen Y, Almirall-Sanchez A, Higgins D, Galvin M, Kane J, et al. Care burden, loneliness, and social isolation in caregivers of people with physical and brain health conditions in English-speaking regions: Before and during the COVID-19 pandemic. Int J Geriatr Psychiatry. 2022;37(6):10.1002/gps.5734. doi: 10.1002/gps.5734 35574817 PMC9324775

[9] Thrush A, Hyder AA. The neglected burden of caregiving in low- and middle-income countries. Disabil Health J. 2014;7(3):262–72. doi: 10.1016/j.dhjo.2014.01.003 24947567

[10] Lawn S, McMillan J, Comley C. Support needs of family caregivers of mental health consumers: a qualitative study. Int J Ment Health Nurs. 2017;26(5):454–62.

[11] Wang T, Molassiotis A, Chung BPM, Tan J-Y. Unmet care needs of advanced cancer patients and their informal caregivers: a systematic review. BMC Palliat Care. 2018;17(1):96. doi: 10.1186/s12904-018-0346-9 30037346 PMC6057056

[12] Freedman VA, Agree EM, Seltzer JA, Birditt KS, Fingerman KL, Friedman EM, et al. The Changing Demography of Late-Life Family Caregiving: A Research Agenda to Understand Future Care Networks for an Aging U.S. Population. Gerontologist. 2024;64(2):gnad036. doi: 10.1093/geront/gnad036 36999951 PMC10825830

[13] Hengelaar AH, Wittenberg Y, Kwekkeboom R, Van Hartingsveldt M, Verdonk P. Intersectionality in informal care research: a scoping review. Scand J Public Health. 2023;51(1):106–24. doi: 10.1177/14034948211027816 34232094 PMC9903248

[14] Prince M, Guerchet M, Prina M. The epidemiology and impact of dementia—current state and future trends. WHO thematic briefing. 2015. Available from: https://hal.science/hal-03517019/document

[15] Wimo A, Seeher K, Cataldi R, Cyhlarova E, Dielemann JL, Frisell O, et al. The worldwide costs of dementia in 2019. Alzheimers Dement. 2023;19(7):2865–73. doi: 10.1002/alz.12901 36617519 PMC10842637

[16] Beach B, Bélanger-Hardy L, Harding S, Rodrigues Perracini M, Garcia L, Tripathi I, et al. Caring for the caregiver: Why policy must shift from addressing needs to enabling caregivers to flourish. Front Public Health. 2022;10:997981. doi: 10.3389/fpubh.2022.997981 36339159 PMC9626797

[17] Sabo K, Chin E. Self-care needs and practices for the older adult caregiver: An integrative review. Geriatr Nurs. 2021;42(2):570–81. doi: 10.1016/j.gerinurse.2020.10.013 33160748

[18] Morse D, Sandhu S, Mulligan K, Tierney S, Polley M, Chiva Giurca B. Global developments in social prescribing. BMJ Glob Health. 2022;7(5):e008524. 10.1136/bmjgh-2022-008524 PMC9115027 35577392

[19] Tierney S, Wong G, Roberts N, Boylan A-M, Park S, Abrams R, et al. Supporting social prescribing in primary care by linking people to local assets: a realist review. BMC Med. 2020;18(1):49. doi: 10.1186/s12916-020-1510-7 32164681 PMC7068902

[20] Pescheny JV, Randhawa G, Pappas Y. The impact of social prescribing services on service users: a systematic review of the evidence. Eur J Public Health. 2020;30(4):664–73. doi: 10.1093/eurpub/ckz078 31199436

[21] Muhl C, Mulligan K, Bayoumi I, Ashcroft R, Godfrey C. Establishing internationally accepted conceptual and operational definitions of social prescribing through expert consensus: a Delphi study. BMJ Open. 2023;13(7):e070184. doi: 10.1136/bmjopen-2022-070184 37451718 PMC10351285

[22] NHS England. Social prescribing [Internet]. NHS England. Available from: https://www.england.nhs.uk/personalisedcare/social-prescribing/

[23] Liebmann M, Pitman A, Hsueh Y-C, Bertotti M, Pearce E. Do people perceive benefits in the use of social prescribing to address loneliness and/or social isolation? A qualitative meta-synthesis of the literature. BMC Health Serv Res. 2022;22(1):1264. doi: 10.1186/s12913-022-08656-1 36261835 PMC9580419

[24] Stickley T, Hui A. Social prescribing through arts on prescription in a U.K. city: participants’ perspectives (part 1). Public Health. 2012;126(7):574–9. doi: 10.1016/j.puhe.2012.04.002 22683358

[25] Wakefield JRH, Kellezi B, Stevenson C, McNamara N, Bowe M, Wilson I, et al. Social Prescribing as “Social Cure”: A longitudinal study of the health benefits of social connectedness within a Social Prescribing pathway. J Health Psychol. 2022;27(2):386–96. doi: 10.1177/1359105320944991 32700974 PMC8793307

[26] White C, Bell J, Reid M, Dyson J. More than signposting: Findings from an evaluation of a social prescribing service. Health Soc Care Community. 2022;30(6):e5105–14. doi: 10.1111/hsc.13925 35915879

[27] Kadri Z. Social prescribing for unpaid caregivers: a scoping review protocol [Internet]. OSF; 2024. Available from: https://osf.io/dgr8p 10.1371/journal.pone.0347299 PMC13098922 42013136

[28] Arksey H, O’Malley L. Scoping studies: towards a methodological framework. Int J Soc Res Methodol. 2005;8(1):19–32.

[29] Peters MDJ, Marnie C, Tricco AC, Pollock D, Munn Z, Alexander L, et al. Updated methodological guidance for the conduct of scoping reviews. JBI Evid Implement. 2021;19(1):3–10. doi: 10.1097/XEB.0000000000000277 33570328

[30] Pollock D, Peters MDJ, Khalil H, McInerney P, Alexander L, Tricco AC, et al. Recommendations for the extraction, analysis, and presentation of results in scoping reviews. JBI Evid Synth. 2023;21(3):520–32. doi: 10.11124/JBIES-22-00123 36081365

[31] Tricco AC, Lillie E, Zarin W, O’Brien KK, Colquhoun H, Levac D, et al. PRISMA Extension for Scoping Reviews (PRISMA-ScR): Checklist and Explanation. Ann Intern Med. 2018;169(7):467–73. doi: 10.7326/M18-0850 30178033

[32] Bickerdike L, Booth A, Wilson PM, Farley K, Wright K. Social prescribing: less rhetoric and more reality. A systematic review of the evidence. BMJ Open. 2017;7(4):e013384. doi: 10.1136/bmjopen-2016-013384 28389486 PMC5558801

[33] Vidovic D, Reinhardt GY, Hammerton C. Can Social Prescribing Foster Individual and Community Well-Being? A Systematic Review of the Evidence. Int J Environ Res Public Health. 2021;18(10):5276. doi: 10.3390/ijerph18105276 34063543 PMC8156788

[34] Covidence systematic review software [Internet]. Melbourne (AU): Veritas Health Innovation. Available from: www.covidence.org

[35] Clarke V, Braun V. Thematic analysis. In: Michalos AC, editor. Encyclopedia of quality of life and well-being research. Dordrecht: Springer; 2014. p. 6626–8.

[36] Baker FA, Stretton-Smith P, Clark IN, Tamplin J, Lee Y-EC. A Group Therapeutic Songwriting Intervention for Family Caregivers of People Living With Dementia: A Feasibility Study With Thematic Analysis. Front Med (Lausanne). 2018;5:151. doi: 10.3389/fmed.2018.00151 29872659 PMC5972290

[37] Burnside LD, Knecht MJ, Hopley EK, Logsdon RG. here:now - Conceptual model of the impact of an experiential arts program on persons with dementia and their care partners. Dementia (London). 2017;16(1):29–45. doi: 10.1177/1471301215577220 25795584

[38] Fancourt D, Warran K, Finn S, Wiseman T. Psychosocial singing interventions for the mental health and well-being of family carers of patients with cancer: results from a longitudinal controlled study. BMJ Open. 2019;9(8):e026995. doi: 10.1136/bmjopen-2018-026995 31401592 PMC6701813

[39] Flatt JD, Liptak A, Oakley MA, Gogan J, Varner T, Lingler JH. Subjective experiences of an art museum engagement activity for persons with early-stage Alzheimer’s disease and their family caregivers. Am J Alzheimers Dis Other Demen. 2015;30(4):380–9. doi: 10.1177/1533317514549953 25216658 PMC4362745

[40] McGuigan KA, Legget JA, Horsburgh M. Visiting the museum together: Evaluating a programme at Auckland Museum for people living with dementia and their carers. Arts & Health. 2015;7(3):261–70. doi: 10.1080/17533015.2015.1045531

[41] Mittelman MS, Papayannopoulou PM. The Unforgettables: a chorus for people with dementia with their family members and friends. Int Psychogeriatr. 2018;30(6):779–89. doi: 10.1017/S1041610217001867 29375037

[42] Pienaar L, Reynolds F. “A respite thing”: A qualitative study of a creative arts leisure programme for family caregivers of people with dementia. Health Psychol Open. 2015;2(1):2055102915581563. doi: 10.1177/2055102915581563 28070356 PMC5193266

[43] Razani N, Morshed S, Kohn MA, Wells NM, Thompson D, Alqassari M, et al. Effect of park prescriptions with and without group visits to parks on stress reduction in low-income parents: SHINE randomized trial. PLoS One. 2018;13(2):e0192921. doi: 10.1371/journal.pone.0192921 29447248 PMC5814008

[44] Baik H, Choi S, An M, Jin H, Kang I, Yoon W, et al. Effect of Therapeutic Gardening Program in Urban Gardens on the Mental Health of Children and Their Caregivers with Atopic Dermatitis. Healthcare (Basel). 2024;12(9):919. doi: 10.3390/healthcare12090919 38727476 PMC11083003

[45] Donnelly KZ, Baker K, Pierce R, St Ivany AR, Barr PJ, Bruce ML. A retrospective study on the acceptability, feasibility, and effectiveness of LoveYourBrain Yoga for people with traumatic brain injury and caregivers. Disabil Rehabil. 2021;43(12):1764–75. doi: 10.1080/09638288.2019.1672109 31577456

[46] Dorard G, Vioulac C, Mathieu S, Ellien F, Bourgeois A, Untas A. Profiles of French young carers taking part in an arts and respite care program. Health Soc Care Community. 2022;30(5):e3253–64. doi: 10.1111/hsc.13769 35199897 PMC9539863

[47] Giebel C, Morley N, Komuravelli A. A socially prescribed community service for people living with dementia and family carers and its long-term effects on well-being. Health Soc Care Community. 2021;29(6):1852–7. doi: 10.1111/hsc.13297 33528081

[48] Girdler M, Fletcher P, Bryden P. An examination of the lived experiences of individuals with dementia and their caregivers in the Minds in Motion® program. Palaestra. 2024;38(1).

[49] Lee S, O’Neill D, Moss H. Promoting well-being among people with early-stage dementia and their family carers through community-based group singing: a phenomenological study. Arts Health. 2022;14(1):85–101. doi: 10.1080/17533015.2020.1839776 33119993

[50] McManus K, Tao H, Jennelle PJ, Wheeler JC, Anderson GA. The effect of a performing arts intervention on caregivers of people with mild to moderately severe dementia. Aging Ment Health. 2022;26(4):735–44. doi: 10.1080/13607863.2021.1891200 33769137

[51] Sun W, Bartfay E, Smye V, Biswas S, Newton D, Pepin M, et al. Living well with dementia: The role volunteer-based social recreational programs in promoting social connectedness of people with dementia and their caregivers. Aging Ment Health. 2022;26(10):1949–62. doi: 10.1080/13607863.2021.1950614 34353187

[52] Vaajoki A, Turjamaa R, Lakka T, Mäkinen E, Välimäki T. A participatory arts programme - Shared experience for family caregivers and care recipients. Nurs Open. 2023;10(5):3011–7. doi: 10.1002/nop2.1547 36504364 PMC10077393

[53] Kim B, Wister A, O’dea E, Mitchell BA, Li L, Kadowaki L. Roles and experiences of informal caregivers of older adults in community and healthcare system navigation: a scoping review. BMJ Open. 2023;13(12):e077641. doi: 10.1136/bmjopen-2023-077641 38070939 PMC10729038

[54] McGhan G, McCaughey D, Flemons K, Shapkin K, Parmar J, Anderson S, et al. Tailored, Community-Based Programs for People Living With Dementia and Their Family Caregiver. J Gerontol Nurs. 2022;48(4):26–32. doi: 10.3928/00989134-20220401-06 35343837

[55] Brooks D, Fielding E, Beattie E, Edwards H, Hines S. Effectiveness of psychosocial interventions on the psychological health and emotional well-being of family carers of people with dementia following residential care placement: a systematic review. JBI Database System Rev Implement Rep. 2018;16(5):1240–68. doi: 10.11124/JBISRIR-2017-003634 29762315

[56] Grover S, Sandhu P, Nijjar GS, Percival A, Chudyk AM, Liang J, et al. Older adults and social prescribing experience, outcomes, and processes: a meta-aggregation systematic review. Public Health. 2023;218:197–207. doi: 10.1016/j.puhe.2023.02.016 37060740

[57] Percival A, Newton C, Mulligan K, Petrella R, Ashe M. Systematic review of social prescribing and older adults: where to from here? Fam Med Community Health. 2022;10(Suppl 1):e001640. 10.1136/fmch-2022-001829 PMC9557282 36207017

[58] Cohen SA, Sabik NJ, Cook SK, Azzoli AB, Mendez-Luck CA. Differences within Differences: Gender Inequalities in Caregiving Intensity Vary by Race and Ethnicity in Informal Caregivers. J Cross Cult Gerontol. 2019;34(3):245–63. doi: 10.1007/s10823-019-09381-9 31407137

[59] Kindratt TB, Sylvers DL, Yoshikawa A, López-Anuarbe M, Webster NJ, Bouldin ED. Dementia Caregiving Experiences and Health Across Geographic Contexts by Race and Ethnicity. J Gerontol B Psychol Sci Soc Sci. 2023;78(Suppl 1):S48–58. doi: 10.1093/geronb/gbac182 36913373 PMC10010466

[60] Liu C, Badana ANS, Burgdorf J, Fabius CD, Roth DL, Haley WE. Systematic Review and Meta-Analysis of Racial and Ethnic Differences in Dementia Caregivers’ Well-Being. Gerontologist. 2021;61(5):e228–43. doi: 10.1093/geront/gnaa028 32271380 PMC8276619

[61] Ditton A, Alodan H, Fox C, Evans S, Cross J. Exploring the effectiveness and experiences of people living with dementia interacting with digital interventions: A mixed methods systematic review. Dementia (London). 2025;24(3):506–51. doi: 10.1177/14713012241302371 39604136 PMC11915779

[62] Marshall J, Papavasiliou E, Allan L, Bradbury K, Fox C, Hawkes M, et al. Reimagining Dementia Care: A Complex Intervention Systematic Review on Optimising Social Prescribing (SP) for Carers of People Living With Dementia (PLWD) in the United Kingdom. Health Expect. 2025;28(3):e70286. doi: 10.1111/hex.70286 40346943 PMC12064994

