# Supplementary material for: Social prescribing programs involving unpaid caregivers: A scoping review
Source: PLoS One. 2026 Apr 21;21(4):e0347299. doi: 10.1371/journal.pone.0347299 (PMC13098922; doi:10.1371/journal.pone.0347299)
Supplement: S2 File — (DOCX) [file pone.0347299.s002.docx]

# **Sample Search Strategy**

**Database:**
OVID Medline Epub Ahead of Print, In-Process & Other Non-Indexed Citations, Ovid MEDLINE(R) Daily and Ovid MEDLINE(R) 1946 to Present

| **#** | **Query** |
| --- | --- |
| 1 | Caregivers/ |
| 2 | carer*.mp. |
| 3 | caregiver*.mp. |
| 4 | 1 or 2 or 3 |
| 5 | social* prescri*.mp. |
| 6 | non medical refer*.mp. |
| 7 | (refer* model* adj2 community).mp. |
| 8 | (refer* adj2 community).mp. |
| 9 | Community Participation/ |
| 10 | Social Participation/ |
| 11 | "Referral and Consultation"/ |
| 12 | 9 or 10 |
| 13 | 11 and 12 |
| 14 | green referral*.mp. |
| 15 | blue referral*.mp. |
| 16 | green prescri*.mp. |
| 17 | blue prescri*.mp. |
| 18 | social* refer*.mp. |
| 19 | link worker*.mp. |
| 20 | exercise referral*.mp. |
| 21 | exercise prescri*.mp. |
| 22 | (prescri* adj2 communit*).mp. |
| 23 | art prescri*.mp. |
| 24 | art referral*.mp. |
| 25 | ecotherap*.mp. |
| 26 | community connector*.mp. |
| 27 | social cafe*.mp. |
| 28 | link coordinat*.mp. |
| 29 | community navigator*.mp. |
| 30 | System Navigator*.mp. |
| 31 | prescri* pathway*.mp. |
| 32 | Patient Navigation/ |
| 33 | (signpost* or sign post*).mp. [mp=title, book title, abstract, original title, name of substance word, subject heading word, floating sub-heading word, keyword heading word, organism supplementary concept word, protocol supplementary concept word, rare disease supplementary concept word, unique identifier, synonyms, population supplementary concept word, anatomy supplementary concept word] |
| 34 | non medical referral*.mp. |
| 35 | ((natur* or pet or yoga or mindfulness or dance or spirit* or forest* or park*) adj2 (prescri* or referral*)).mp. [mp=title, book title, abstract, original title, name of substance word, subject heading word, floating sub-heading word, keyword heading word, organism supplementary concept word, protocol supplementary concept word, rare disease supplementary concept word, unique identifier, synonyms, population supplementary concept word, anatomy supplementary concept word] |
| 36 | social support intervention*.mp. |
| 37 | ((individual* or tailor*) adj (program* or intervention* or service*)).mp. |
| 38 | (participat* adj program*).mp. [mp=title, book title, abstract, original title, name of substance word, subject heading word, floating sub-heading word, keyword heading word, organism supplementary concept word, protocol supplementary concept word, rare disease supplementary concept word, unique identifier, synonyms, population supplementary concept word, anatomy supplementary concept word] |
| 39 | (co design* or co produc* or co creat*).mp. |
| 40 | (navigat* adj3 (program* or intervention* or service*)).mp. |
| 41 | navigator*.mp. |
| 42 | social [group.mp](http://group.mp). |
| 43 | (referral* adj intervention*).mp. |
| 44 | (park* adj2 prescri*).mp. [mp=title, book title, abstract, original title, name of substance word, subject heading word, floating sub-heading word, keyword heading word, organism supplementary concept word, protocol supplementary concept word, rare disease supplementary concept word, unique identifier, synonyms, population supplementary concept word, anatomy supplementary concept word] |
| 45 | (forest adj (therapy* or prescri*)).mp. |
| 46 | ((well-being or well-being) adj (program* or intervention*)).mp. |
| 47 | (commun* adj2 (program* or group* or prescri* or referral*)).mp. [mp=title, book title, abstract, original title, name of substance word, subject heading word, floating sub-heading word, keyword heading word, organism supplementary concept word, protocol supplementary concept word, rare disease supplementary concept word, unique identifier, synonyms, population supplementary concept word, anatomy supplementary concept word] |
| 48 | Health Promotion/ and Community Health Services/ |
| 49 | social cafe*.mp. |
| 50 | (statutory adj (service* or program* or intervention*)).mp. |
| 51 | ((health* or commun*) adj (navigator* or aide* or advisor* or co-ordinator* or coordinator* or connector* or officer* or manager* or facilitator? or liaison or coach or promoter*)).mp. [mp=title, book title, abstract, original title, name of substance word, subject heading word, floating sub-heading word, keyword heading word, organism supplementary concept word, protocol supplementary concept word, rare disease supplementary concept word, unique identifier, synonyms, population supplementary concept word, anatomy supplementary concept word] |
| 52 | ((art* or cultur* or cook* or music* or horticultur* or religi* or garden*) adj (prescri* or refer* or program*)).mp. [mp=title, book title, abstract, original title, name of substance word, subject heading word, floating sub-heading word, keyword heading word, organism supplementary concept word, protocol supplementary concept word, rare disease supplementary concept word, unique identifier, synonyms, population supplementary concept word, anatomy supplementary concept word] |
| 53 | ((social determinant* or social risk* or social need* or holistic need*) and (connect* or refer* or navigator* or link* or prescri*)).mp. [mp=title, book title, abstract, original title, name of substance word, subject heading word, floating sub-heading word, keyword heading word, organism supplementary concept word, protocol supplementary concept word, rare disease supplementary concept word, unique identifier, synonyms, population supplementary concept word, anatomy supplementary concept word] |
| 54 | (non clinical adj (refer* or program* or prescri*)).mp. [mp=title, book title, abstract, original title, name of substance word, subject heading word, floating sub-heading word, keyword heading word, organism supplementary concept word, protocol supplementary concept word, rare disease supplementary concept word, unique identifier, synonyms, population supplementary concept word, anatomy supplementary concept word] |
| 55 | 11 and 48 |
| 56 | ((individual* or tailor*) adj (program* or intervention* or service*)).mp. |
| 57 | 5 or 6 or 7 or 8 or 13 or 14 or 15 or 16 or 17 or 18 or 19 or 20 or 21 or 22 or 23 or 24 or 25 or 26 or 27 or 28 or 29 or 30 or 31 or 32 or 33 or 34 or 35 or 36 or 37 or 38 or 39 or 40 or 41 or 42 or 43 or 44 or 45 or 46 or 47 or 49 or 50 or 51 or 52 or 53 or 54 or 56 |
| 58 | 4 and 57 |
| 59 | limit 58 to yr="2000 -Current" |
| 60 | limit 58 to yr="2000 -Current" |
| 61 | limit 60 to english language |
